# Supplementary material for: A community health worker-led program to improve access to gestational diabetes screening in urban slums of Pune, India: Results from a mixed methods study
Source: PLOS Glob Public Health. 2023 Oct 27;3(10):e0001622. doi: 10.1371/journal.pgph.0001622 (PMC10610081; doi:10.1371/journal.pgph.0001622)
Supplement: S1 Table — (DOCX) [file pgph.0001622.s001.docx]

| **S1 Table.** Characteristics of women who underwent OGTT | |
| --- | --- |
| **Characteristic** | **Total (n=223)**  **N (%)** |
| Age, median [IQR] | 24 (22-27) |
| Gestational age, weeks, median (IQR) | 26 (24-31) |
| Known family history of diabetes | 27 (12%) |
| High blood pressure (>130/80 mmHg) | 28 (13%) |
| High school diploma or greater | 106 (47.9%) |
| BMI, kg/m^2^, median (IQR) | 23.8 (21.4-27.4) |
| Mid-upper arm circumference, cm, (IQR) | 26 (24-29) |
| Waist circumference, cm, (IQR) | 96 (90-103) |
| Diagnosed with GDM | 31 (14%) |
| BMI, kg/m^2^, median (IQR) for women diagnosed with GDM | 25.5 (22.0-29.3) |
| Kuppuswamy socioeconomic status^25^ | |
| *Upper-middle income* | 13 (6%) |
| *Lower-middle income* | 118 (53%) |
| *Upper-lower income* | 92 (41%) |
