# Supplementary material for: A community health worker-led program to improve access to gestational diabetes screening in urban slums of Pune, India: Results from a mixed methods study
Source: PLOS Glob Public Health. 2023 Oct 27;3(10):e0001622. doi: 10.1371/journal.pgph.0001622 (PMC10610081; doi:10.1371/journal.pgph.0001622)
Supplement: S1 Text — (DOCX) [file pgph.0001622.s002.docx]

**S1 Text. Interview guide for participants**

1. Please tell me about your experience participating in this study (Prompts: What happened during your CHW visit when you were enrolled in the study? What was your experience receiving an OGTT and finger stick testing from the CHW? Did you find the CHWs’ instructions easy to follow?)

2. After receiving your OGTT results, please tell me what happened. (Prompts: How did you feel about your health after your CHW visit? How did you feel about your baby’s health after your CHW visit?)

- If screened GDM positive: Please tell me about what happened after you were referred to the prenatal clinic for further care? (Prompts: Did you seek additional care? Why/why not?)
- For those who received clinic-based GDM care/treatment: Please tell me about your experience receiving care at the clinic. (Prompts: How long did you wait for your confirmatory test and treatment? How did you travel to the clinic? Did you feel comfortable going there for care? Why/Why not? Did you like the healthcare provider? How have the GDM test results impacted your life since then?)
- For those who did **not** receive GDM care/treatment: Please tell me about your decision to not receive further care. (Prompts: What were the most important factors that led you to not complete the referral for GDM care? How do you think that GDM will affect your life, if at all?)

3. How has participation in this study affected your thoughts about gestational diabetes? (Prompts: Have you discussed anything with friends or family about gestational diabetes? Do you know other people with gestational diabetes? Do you think gestational diabetes is dangerous? Why/why not?)

4. Please tell me about any difficulties, if any, you experienced receiving prenatal care in general during your current pregnancy (Prompts: barriers may include time, transportation, illness, competing priorities, COVID-related lockdown)

Version Date: 6/8/21
