# Supplementary material for: A community health worker-led program to improve access to gestational diabetes screening in urban slums of Pune, India: Results from a mixed methods study
Source: PLOS Glob Public Health. 2023 Oct 27;3(10):e0001622. doi: 10.1371/journal.pgph.0001622 (PMC10610081; doi:10.1371/journal.pgph.0001622)
Supplement: S2 Text — (DOCX) [file pgph.0001622.s003.docx]

**S2 Text. Interview guide for CHWs**

1. Please tell me about your experience participating in this study. (Prompts: Has this work changed your thoughts about GDM? Do you think gestational diabetes is dangerous? Why/ why not? Did you feel the work was effective at helping pregnant women? Why/why not?)

2. Please tell me about your experiences during the GDM training sessions. (Prompts: Was there anything you wish you learned more about? Did the training prepare you for the work in the community? Why/why not?)

3. What was your experience like in delivering OGTT to your patients? (Prompts: How comfortable were you when delivering care instructions? How comfortable did you feel administering OGTTs and checking fingerstick glucose levels? What do you think worked well? What did not work well? Is there an interaction with one participant that stands out in your memory? What happened with that particular woman?)

4. Why do you think some of the women who screened positive for GDM did not receive follow-up at the prenatal clinic? (Prompts: Can you tell us about a participant who did not receive prenatal follow up after the OGTT? What happened in that case? What were the specific barriers for that participant [low perceived need, lack of time/funds/transportation, COVID-related factors])?).

5. Are there other ways you can think of for CHWs to support pregnant women in these communities? Please tell me more.

Version Date: 6/8/21
