# Supplementary material for: A community health worker-led program to improve access to gestational diabetes screening in urban slums of Pune, India: Results from a mixed methods study
Source: PLOS Glob Public Health. 2023 Oct 27;3(10):e0001622. doi: 10.1371/journal.pgph.0001622 (PMC10610081; doi:10.1371/journal.pgph.0001622)
Supplement: Attachment — Submitted filename: Response to Reviewers.pdf [file pgph.0001622.s004.pdf]

Weill Cornell Medicine   NewYork-Presbyterian

Puja Chebrolu, MD
Assistant Professor, Department of Medicine
Center for Global Health
402 E 67th Street, 2nd floor
New York, NY 10065

January 11, 2023

Dear Editorial Team,

We thank you for your initial review of our manuscript and have provided responses to your queries below.

1. Data from this study contain potentially identifying and sensitive patient information. Deidentified data used for this analysis will be made available upon request after signing a data access and use agreement, provision of approval by the SAHARA ethics board, and demonstration that the external investigative team is qualified and has documented evidence of human research protection training. Requests may be addressed to the corresponding author or to irb@med.cornell.edu. For the purpose of peer review only, we have attached 3 fully de-identified interview transcripts to this resubmission.
2. Thank you for bringing this omission to our attention. We obtained written informed consent from all participants. We have added this to the ethics statement within the manuscript.

Sincerely,

Puja Chebrolu, MD
Assistant Professor of Medicine
Weill Cornell Medicine

402 East 67th Street, 2nd floor, New York, NY 10065 | T. 646.965.4423 | F. 646.962.0285 | puc9005@med.cornell.edu
