# Supplementary material for: A community health worker-led program to improve access to gestational diabetes screening in urban slums of Pune, India: Results from a mixed methods study
Source: PLOS Glob Public Health. 2023 Oct 27;3(10):e0001622. doi: 10.1371/journal.pgph.0001622 (PMC10610081; doi:10.1371/journal.pgph.0001622)
Supplement: Attachment — Submitted filename: Reviewer Responses_4.17.23 Final.pdf [file pgph.0001622.s005.pdf]

Weill Cornell Medicine NewYork-Presbyterian

Puja Chebrolu, MD
Assistant Professor, Department of Medicine
Center for Global Health
402 E 67th Street, 2nd floor
New York, NY 10065

April 17, 2023

Dr Senjuti Saha
Guest Editor
PLOS Global Public Health

Dear Dr Saha,

Thank you for providing us the opportunity to revise this manuscript, “A community health worker-led program to improve access to gestational diabetes screening in urban slums of Pune, India: results from a mixed methods study” (PGPH-D-22-02116).

We hope you will find our revision responsive to reviewer comments and look forward to working with your team to publish this work. Please do not hesitate to contact me with any questions.

Sincerely,

Puja Chebrolu, MD
Assistant Professor of Medicine
Weill Cornell Medicine

402 East 67th Street, 2nd floor, New York, NY 10065 | T. 646.965.4423 | F. 646.962.0285 | puc9005@med.cornell.edu

Editor Comments

1. Please send a completed 'Competing Interests' statement, including any COIs declared by your co-authors. If you have no competing interests to declare, please state "The authors have declared that no competing interests exist". Otherwise please declare all competing interests beginning with the statement "I have read the journal's policy and the authors of this manuscript have the following competing interests:"

Response: This has been added to the submission

2. Please amend your detailed Financial Disclosure statement. This is published with the article. It must therefore be completed in full sentences and contain the exact wording you wish to be published.

a. State what role the funders took in the study. If the funders had no role in your study, please state: “The funders had no role in study design, data collection and analysis, decision to publish, or preparation of the manuscript.”
b. If any authors received a salary from any of your funders, please state which authors and which funders.

If you did not receive any funding for this study, please simply state: “The authors received no specific funding for this work.”

Response: This has been added to the manuscript and financial disclosure statement.

3. We do not publish any copyright or trademark symbols that usually accompany proprietary names, eg ©, ®, ™ (e.g. next to drug or reagent names). Please remove all instances of trademark/copyright symbols throughout the text, including ® on page 4.

Response: This has been removed from the manuscript.

4. Fig 1: please (a) provide a direct link to the base layer of the map (i.e., the country or region border shape) and ensure this is also included in the figure legend; and (b) provide a link to the terms of use / license information for the base layer image or shapefile. We cannot publish proprietary or copyrighted maps (e.g. Google Maps, Mapquest) and the terms of use for your map base layer must be compatible with our CC-BY 4.0 license.

Note: if you created the map in a software program like R or ArcGIS, please locate and indicate the source of the basemap shapefile onto which data has been plotted.

If your map was obtained from a copyrighted source please amend the figure so that the base map used is from an openly available source. Alternatively, please provide explicit written permission from the copyright holder granting you the right to publish the material under our CC-BY 4.0 license.

Please note that the following CC BY licenses are compatible with PLOS license: CC BY 4.0, CC BY 2.0 and CC BY 3.0, meanwhile such licenses as CC BY-ND 3.0 and others are not compatible due to additional restrictions.

If you are unsure whether you can use a map or not, please do reach out and we will be able to help you. The following websites are good examples of where you can source open access or public domain maps:
* U.S. Geological Survey (USGS) - All maps are in the public domain. (http://www.usgs.gov)
* PlaniGlobe - All maps are published under a Creative Commons license so please cite "PlaniGlobe, http://www.planiglobe.com, CC BY 2.0" in the image credit after the caption. (http://www.planiglobe.com/?lang=enl)
* Natural Earth - All maps are public domain. (http://www.naturalearthdata.com/about/terms-of-use/)

Response: Open Street Map was used to create the map. Their logo has been displayed on the figure per requirements. The link to the base map is: https://openstreetmap.in/#3.91/22.82/82. The copyright terms are listed here: https://www.openstreetmap.org/copyright

5. We would expect qualitative and mixed-methods studies to include the following:
   1) defined objectives or research questions;
   2) description of the sampling strategy, including rationale for the recruitment method, participant inclusion/exclusion criteria and the number of participants recruited;
   3) detailed reporting of the data collection procedures;
   4) data analysis procedures described in sufficient detail to enable replication;
   5) a discussion of potential sources of bias; and
   6) a discussion of limitations.

   Response: We have added details of points 1) through 4) to the methods section and 5) to 6) to the discussion. In addition, we have added a reference to detail the framework approach we used for data analysis.
   Line numbers: 177-178, 181-184, 186-201, 224-231, 443-447

Reviewer Comments

1. I'm not sure that WHO does in fact recommend all women for screening for GDM by OGTT, they certainly suggest maternal assessment for hyperglycaemia, however in your ref #4 the suggestion is that GDM can be diagnosed with a FPG > 5.1 mmol/L or the two hr OGTT, before 24 – 28 weeks (so late 2nd early 3rd trimester) so I am suggesting this be toned down a bit to truly reflect the WHO guidelines noting as well that WHO do in fact recommend task shifting health promotion, nutritional supplements and IPTp, which you could argue your study fulfills these recommendations by WHO for task shifting see anc-guideline-presentationb8005106-0947-4329-adb1-61aea46db1db.pptx (live.com)

   Response: We appreciate this insight and the link to WHO Antenatal Care guidelines. We have revised the introduction to reflect WHO diabetes screening guidelines as suggested and clarified the differences between gestational diabetes screening recommendations and the task shifting recommendations from WHO. We have noted that task shifting is recommended by the WHO in pregnancy to improve the utilization of antenatal care and that the use of CHWs for GDM screening falls in line with this recommendation.
   Line numbers: 74-76, 86-88

2. The description of sampling and recruitment requires a bit more detail, how big were the study areas, did they go door to door to every single dwelling in the study areas or was there a more household sampling approach, what was the denominator population (ie women of CBA, or some way to estimate reach of the CHW recruitment)

    Response: We have added details about the study population and recruitment strategies used for the study. For the denominator population, we used birth rate data from Maharashtra to estimate the number of pregnant women in each area.
    Line numbers: 118-121

3. How were the CHW trained to collect anthropometry and BP as these are skills that need to be acquired after appropriate training – seems like they only received training for the OGTT, what were there skill levels for other clinical assessments – were they ASHA, or angawadi or some other specific cadre. How were they trained to deliver counselling about healthy diet etc where did this health promotion information come from or was it developed for the study

    Response: The CHW were trained by an obstetric nurse on how to accurately measure anthropometrics and vital signs for pregnant women. The nurse followed Federation of Obstetric and Gynaecological Societies in India (FOGSI) guidelines to provide education on GDM diet and lifestyle counseling. We produced an informational flyer summarizing guidelines that was also provided to participants with GDM. We have added details about training and counseling to the manuscript.
    Line numbers: 140-146

4. How was gestational age assessed during the recruitment process to determine the woman was more than 24 weeks pregnant.

    Response: CHWs were trained to use a pregnancy wheel to assess gestational age based on reported last menstrual period or by sonographic dates when this information was available.
    Line numbers: 125-17

5. How was BMI calculated during the pregnancy? It is not as simple as for non-pregnant women (wgt/height2)

    Response: We used the standard calculation for BMI (weight divided by height^2) but as the reviewer mentions, there are no standard cutoffs during pregnancy, so we avoided interpretation of BMI by using BMI as a continuous variable. There are cutoffs for mid-upper arm circumference. We have now added mid-upper arm circumference and waist circumference to the table. We have compared these parameters between women with and without GDM.
    Line number: Table 1 and supplementary data

6. How far away were “the nearby antenatal clinic” to which the women were referred. As there is discussion about logistics to get to antenatal care it’s reasonable to want to know how far women might need to travel to access this care (time or distance).

Response: The Sassoon General Hospital antenatal clinic, which is government-run and therefore provides free care, was within 5 kms of both sites. This information has been added to the paper.
Line number: 167

7. Where was the REDCap server and data held, who was the custodian or the data was that the Deep Griha Society, What was the role of this society in the collaboration.

    Response: The REDCap server is maintained by Weill Cornell Medicine, and only study investigators at Deep Griha and Weill Cornell had access to the data. Deep Griha Society (DGS) is a non-governmental organization that has been conducting slum health outreaches for >40 years. DGS hired CHWs for this study. Weill Cornell and DGS collaboratively oversaw CHW training and activities. Weill Cornell oversaw data collection and analysis.
    Line numbers: 136-138

8. What measures were taken to ensure the safety of the water in which the OGTT was prepared?, was this just regular household drinking water?

    Response: In this study, the CHWs were provided bottled water to take to each OGTT. For implementation of this intervention on a larger scale, the type of water provided may be an important consideration
    Line number: 152

9. What was the actual referral process? Were they just advised to go to the clinic or were they given appointments or letters of referral for the health centre/clinic.

    Response: Thank you for this question. The referral process was as follows. Women who met GDM criteria via OGTT or had abnormal vital signs were provided a referral letter with the details of their test results and advised to go to the clinic. No appointments were given as there is no appointment system at the referral hospital. However, they were able to contact the CHW if they needed assistance navigating the hospital system. These details have been added to the Methods section
    Line number: 165-167

10. Why was ethics not necessary or obtained from ICMR particularly if it was an overseas based study, having conducted a similar task shifting study in India we were required to obtained ethical approval from ICMR.

    Response: ICMR approval was not deemed necessary for this particular study because the funding for the study (e.g. CHW, interviews, etc) was provided through Deep Griha (the Indian NGO).
    Line number: 172

11. There are no sample/power calculations described, although you mention in the discussion of strengths that it was a well powered study?

Response: Thank you for pointing this out. We calculated sample size a priori based on an estimated uptake of 20%. We have revised the manuscript to include both a priori and post-hoc sample size analyses.
Line number: 202-207

12. Reference required for the SES scale used (Kuppuswamy socioeconomic status)

    Response: We have added a reference to the Kuppuswamy scale.
    Line number: 218

13. 13% of women had hypertension, what was done for these women, were any of these women also GDM?

    Response: Women who met criteria for hypertension during screening were referred to the government clinic which served as the referral center for our study. 7 (23%) women with GDM had hypertension, compared to 21 (11%) women without GDM. The higher prevalence of hypertension among women with GDM is consistent with what has been described in prior literature. We did not include details about hypertension as it was not the primary focus of this manuscript.
    Line number: 165-167, Table 1

14. You state reasons for “eligible” women not completing OGTT was previous OGTT (Figure 3), and yet in the eligibility criteria these women should have been excluded?

    Response: Thank you for the opportunity to clarify this point. The ‘previous OGTT’ we were referring to was a 1st trimester OGTT. Some women underwent a 1st trimester OGTT. First trimester OGTT is diagnostic of pre-pregnancy diabetes and not GDM. Therefore, we did not exclude these women from enrollment. However, several women did not want to repeat the OGTT to screen for GDM. We have added ‘for type 2 diabetes screening’ to the figure to further clarify this.
    Line number: Figure 3

15. You mention several times the “logistics” as a barrier to attending the clinic, but I only read mention of travel, would be good to outline this a bit more, particularly in the context of time and distance (so a bit more explanation about how far the women were from health centres/how much transport might cost)

    Response: We agree that there are several other financial and logistic barriers to OGTT screening. We have added discussion of other barriers, including lost wages, cost of travel, other caretaking responsibilities, and time spent in clinic.
    Line number: 77-79

16. I find the quote on line 225 a bit strange, had she had OGTT before how did she know it took all day, how was she aware of the OGTT before the CHW came, did she have a history of GDM?

    Response: This quote by a woman stating that OGTT from a prior pregnancy had previously taken all day. Median age at first birth in India is age 21. Her interview indicates that she had been through OGTT screening before in a clinical setting.

17. Figure 2 study flow chart – you need to describe why the 10% of eligible women were not enrolled in the figure,

    Response: 10% of the women who were eligible declined enrollment. We have updated figure 2 language to clarify this. The reasons for declined enrollment are described in detail in Fig 3. We have added a reference to Fig 3 into Fig 2.
    Line number: Fig 2

18. You detected 14% of women with GDM and describe it as "surprisingly high prevalence" – however in the background (reference #1) you state up to 40% and then in the discussion state this 14% was higher than the previously reported national average (reference #27). These two sentences and references appear inconsistent.

    Response: Thank you for pointing out this discrepancy. We have removed the reference to the 40% prevalence found in one study in a high-income group in India. This data is an outlier when compared to the GDM prevalence in India as a whole. We have now included the national average in India as a reference; it is 9%
    Line number: 66, 378

19. Line 330, the discussion of BMI is a bit confusing whilst the BMI of the entire cohort was 23.8, the median BMI of women with GDM was more than 25.5 which confirms the risk profile. BMI is not the most reliable assessment of risk though.

    Response: As the median BMI is 25.5 kg/m^2, 50% of these women had a BMI <25 kg/m^2 and would have been missed on BMI-based risk assessment. We agree that BMI is not the most reliable assessment of risk, particularly among South Asians. However, most global guidelines include BMI cutoffs rather than abdominal girth/waist circumference. We therefore included this in the discussion to point out that this population would not traditionally be considered high risk for GDM.
    Line number: 399-401

20. In general, the quality of the figures is low, can you provide better quality images?

    Response: We have improved the image quality and resubmitted higher quality images for review.
    Line number: all figures

21. Figure 1. Is it possible perhaps to include health centres in this figure and provide a bit more detail.

    Response: We have included the 'public government hospital', which was the referral centre.
    Line number: Figure 1

22. Figure 2 provide reasons for unable to be enrolled, how many just refused without reason/not interested.

Response: As above, this is described in detail in Figure 3. We have added a reference to Figure 3 in Figure 2.
Line number: Figure 2

23. Figure 3 as previously mentioned the 34.8% with a previous OGTT should have been excluded shouldn't they as they did fit the inclusion criteria

    Response: As in Question 14 above, a 1st trimester OGTT was performed in some cases as a screening for diabetes mellitus (type 2 diabetes), not GDM. We have added 'for type 2 diabetes screening' to the figure to further clarify
    Line number: Figure 3

24. Figure 4 – poor quality difficult to read even when zoomed in numbers are all blurry

    Response: We have improved the image quality and resubmitted higher quality images for review.
    Line number: All figures

25. Supplemental table 1 would be better in the actual manuscript deleting Table 1 currently in main manuscript.

    Response: We have now included supplemental table 1 as table 1 in the main manuscript
    Line number: Table 1

26. You could provide the Interview questions only for the participants/CHW/ and Clinicians, you do not have to provide any responses but it is important to know what questions were asked during the interviews

    Response: We agree that providing the interview questions may help a reader better contextualized our results. We have now provided the interview guides as supplementary files.
    Line number: Supplementary material

27. References: #4 the link provided does not direct the reader to the full article rather a summary. Link should be changed to Diagnostic criteria and classification of hyperglycaemia first detected in pregnancy: A World Health Organization Guideline (diabetesresearchclinicalpractice.com).
    Also if you wish to reference the summary why not use the updated version (august 2021) WHO recommendation on the diagnosis of gestational diabetes in pregnancy (srhr.org)

    Response: Thank you for providing the updated summary. We have replaced this reference.
    Line number: 478
