# Supplementary material for: A community health worker-led program to improve access to gestational diabetes screening in urban slums of Pune, India: Results from a mixed methods study
Source: PLOS Glob Public Health. 2023 Oct 27;3(10):e0001622. doi: 10.1371/journal.pgph.0001622 (PMC10610081; doi:10.1371/journal.pgph.0001622)
Supplement: Attachment — Submitted filename: Reviewer Responses_7.10.23.pdf [file pgph.0001622.s006.pdf]

Puja Chebrolu, MD
Assistant Professor, Department of Medicine
Center for Global Health
402 E 67th Street, 2nd floor
New York, NY 10065

July 10, 2023

Dr Hannah Landenmark
Staff Editor
PLOS Global Public Health

Dear Dr Landenmark,

Thank you for providing us the opportunity to revise this manuscript, "A community health worker-led program to improve access to gestational diabetes screening in urban slums of Pune, India: results from a mixed methods study" (PGPH-D-22-02116).

We particularly thank the new reviewer for insightful comments which have improved the manuscript. We hope you will find our revision responsive to reviewer comments and look forward to working with your team to publish this work. Please do not hesitate to contact me with any questions.

Sincerely,

Puja Chebrolu, MD
Assistant Professor of Medicine
Weill Cornell Medicine

402 East 67th Street, 2nd floor, New York, NY 10065 | T. 646.965.4423 | F. 646.962.0285 | puc9005@med.cornell.edu

1. The authors, in my assessment, have tried their best to address the concerns from the previous round of review. Unfortunately, I was not part of that review team. So, my questions below may appear to be new to the authors. However, I do think that they are important and should be addressed (at least in writing) before the paper can be accepted for publication.

   While I see the benefits of task-shifting and several studies in the past have shown the benefits of this approach across a spectrum of public health contexts and clinical conditions, I am afraid that there is a limit to how much work can be shifted to lay health workers and how that work will be integrated and coordinated.

   In India, we are already have a cadre of community health workers (ASHAs) who are tasked with many activities within maternal and child health domain. One of these tasks include motivating the expectant mother to attend antenatal visit and accompany her for the visit to the health facility. In fact, MoHFW guidelines clearly highlight the goal of universal testing for GDM among all pregnant women and lays out the operational guidelines to achieve this goal including the responsibilities of ASHA workers. See here: https://nhm.gov.in/New_Updates_2018/NHM_Components/RMNCH_MH_Guidelines/Gestational-Diabetes-Mellitus.pdf

   Could the authors comment on how their approach is either consistent with the guidelines or whether their findings imply modification of the guidelines? If the latter, what would be the cost-effectiveness of this approach and how would this approach fit in within the overall workflow of ASHAs with regards to caring for pregnant women? Which of the tasks (other than GDM screening) should be shifted to the community from the health facility? My concern is that, studied in isolation, each task may be better off shifted to the community, but that it may be infeasible to do so when looked at in totality

   Response: Thank you for this insightful comment which considers the feasibility of real-world implementation of this intervention. We agree that incorporating GDM screening into the overall workflow of ASHAs may be infeasible as it may lead to increased task burden for already overworked ASHA workers. However, GDM screening done by an isolated group of CHWs may lead to fragmented care. Among the GDM screening-related tasks that the Ministry of Health delineates for ASHA workers are tasks that require coordination of care between the clinic and the pregnant women. ASHA workers are required to calculate the time at which a woman should be screened, remind her to get the screening done, coordinate with the clinic to arrange a time for screening, accompany the woman to the clinic, and provide counselling on GDM screening results. Our study demonstrates the possibility of streamlining this process if GDM screening was done by the ASHA worker directly. Taken together, adding home OGTT screening to the tasks of the ASHA worker may decrease the burden of her other tasks related to GDM screening while providing gold-standard care to pregnant women. We have added these points to the discussion of the paper. Please see revisions in lines 365-376.
