# Supplementary material for: A community health worker-led program to improve access to gestational diabetes screening in urban slums of Pune, India: Results from a mixed methods study
Source: PLOS Glob Public Health. 2023 Oct 27;3(10):e0001622. doi: 10.1371/journal.pgph.0001622 (PMC10610081; doi:10.1371/journal.pgph.0001622)
Supplement: Attachment — Submitted filename: Reviewer Responses_9.6.23.pdf [file pgph.0001622.s007.pdf]

Weill Cornell Medicine NewYork-Presbyterian

Puja Chebrolu, MD
Assistant Professor, Department of Medicine
Center for Global Health
402 E 67th Street, 2nd floor
New York, NY 10065

September 5, 2023

Dr Sarang Deo
Academic Editor
PLOS Global Public Health

Dear Dr Deo,

Thank you and prior reviewers for providing us the opportunity and impetus to revise this manuscript, "A community health worker-led program to improve access to gestational diabetes screening in urban slums of Pune, India: results from a mixed methods study" (PGPH-D-22-02116) and to think deeply about the real-world implications of this study. We hope you will find our revision responsive to your comments and look forward to working with your team to publish this work. Please do not hesitate to contact me with any questions.

Sincerely,

Puja Chebrolu, MD
Assistant Professor of Medicine
Weill Cornell Medicine

402 East 67th Street, 2nd floor, New York, NY 10065 | T. 646.965.4423 | F. 646.962.0285 | puc9005@med.cornell.edu

1. **Comment:** I see that the authors have attempted to respond to the comments from the new reviewer in the last round. The response and associated edits in the manuscript are mostly satisfactory. However, I would like the authors to add a little more detail on ASHA's ability to conduct OGTT by themselves based on (i) their training and skill level, (ii) government regulations, (iii) willingness of the patient to undergo the test with ASHA. I suggest that the authors look for references in the literature, where similar interventions done by ASHAs are evaluated. I would also like the authors to be less definitive about the time saved of ASHAs if they do the OGTT themselves and explicitly leave it for a future research study to establish this rigorously. I think the paper should be ready for acceptance once these are incorporated in the new draft.

   Response: Thank you for your thoughtful review of our edits and for the framework to improve our discussion of the real-world implications of this study. We have now added information to address each of your points. In particular, we have added (i) ASHA workers training and skill level (young to middle aged women who generally do not have a health background), (ii) government regulations (Ministry of Health guidelines for ASHA workers do not prohibit blood testing), and (iii) the willingness of the patient to undergo testing with the ASHA (we have cited quantitative and mixed methods studies which trained ASHA workers to administer capillary glucose testing for diabetes, with high acceptability and trust). Please see our revisions on lines 366-377.

   We have added several references, particularly of prior interventions employing ASHA workers in capillary glucose testing, and of government of India ASHA guidelines.

   We have also revised the discussion of potential time benefits of ASHA-led testing to indicate that further research should be done to determine whether or not ASHA-led testing would truly be time saving. Please see revision on lines 377-381.
